# Non-invasive techniques to access *in vivo* the skin microcirculation in patients

**DOI:** 10.3389/fmed.2022.1099107

**Published:** 2023-01-05

**Authors:** Daniel Alexandre Bottino, Eliete Bouskela

**Affiliations:** Laboratory for Clinical and Experimental Research on Vascular Biology, Department of Physiological Sciences, State University of Rio de Janeiro, Rio de Janeiro, Brazil

**Keywords:** microcirculation, nailfold videocapillaroscopy, orthogonal polarization spectral imaging (OPS), sidestream dark field imaging, incident dark field illumination, laser Doppler flowmetry, laser speckle contrast imaging

## Abstract

The microcirculation is composed of blood vessels with mean internal diameter smaller than 100 μm. This structure is responsible for survival of cells and in the last 50 years its study has become increasingly interesting because it often participates in the pathophysiology of several diseases or can determine better or worse prognosis for them. Due to the growing importance of knowing more about the microcirculation, several techniques have been developed and now it is possible to study its structure or function. In the last 25 years, the cutaneous microcirculation has emerged as an accessible and representative portion of generalized vascular bed allowing the examination of mechanisms of microcirculatory function and dysfunction. This mini review presents several techniques used for non-invasive access to skin microcirculation, such as Nailfold Videocapillaroscopy, Orthogonal Polarization Spectral Imaging, Sidestream Dark Field Imaging, Incident Dark field Illumination, Laser Doppler Flowmetry, and Laser Speckle Contrast Imaging applied. The techniques presented will describe which types of variables (structural or functional) can be evaluated, their limitations and potential uses.

## Introduction

The microcirculation comprises blood vessels with mean internal diameter smaller than 100 μm. In the last 50 years, its study has become increasingly important due to its involvement in the pathophysiology of several diseases. The microcirculation can often significantly influence the prognosis of patients as well. In the last 25 years, the cutaneous microcirculation has emerged as an accessible and representative portion of generalized vascular bed that can be useful to examine the mechanisms of microcirculatory function and dysfunction (1). As an example, impaired endothelium-dependent vasodilatation can be evidenced by examining the cutaneous microcirculation. It is accepted today that the examination of the cutaneous microcirculation may serve as mirror of systemic vascular dysfunction and underlying mechanisms (2–5).

**FIGURE 1:**
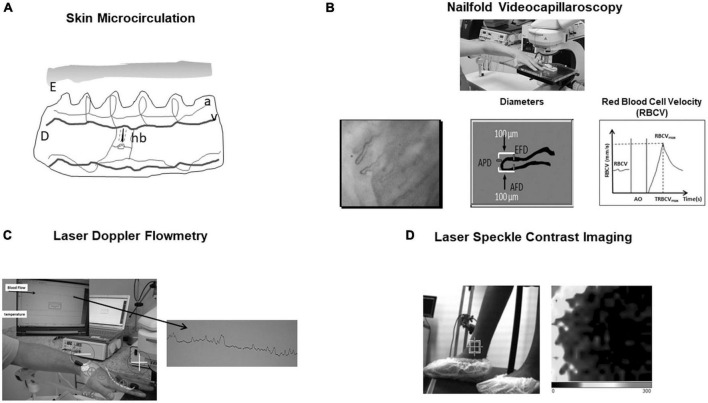
**(A)** Skin microcirculation. E, epidermis; D, dermis; a, arteriole; v, venule; hb, hair bulb. **(B)** Nailfold videocapillaroscopy—One can see how afferent, apical, and efferent capillary diameters (AFD, APD, and EFD) are obtained. Also, baseline Red Blood Cell Velocity (RBCV), RBCV maximum (RBCV_max_), and time to reach RBCV_max_ after arterial occlusion (AO) release are shown. **(C)** Laser Doppler flowmetry. Blood flow is expressed by perfusion index in arbitrary units. Iontophoresis may be associated to evaluate microvascular function. **(D)** Laser speckle contrast imaging. 2-D perfusion maps of tissue surfaces and perfusion in arbitrary units are created. Darker color represents low blood flow and lighter color higher blood flow. Here it can be seen the study of perimaleolar perfusion in chronic venous disease (control patient).

To better understand the study of skin microcirculation we need to describe it. The cutaneous microcirculation has two plexuses: an upper horizontal one with nutritive capillary loops in the dermal papillae orthogonal to the skin accompanied by arteriole/venule and a lower horizontal plexus composed by perforating vessels from underlying muscles and subcutaneous fat. Here there are arterioles and venules that directly connect with the upper horizontal plexus and also provide lateral tributaries that supply hair bulbs and sweat glands (6, 7; Figure 1A).

The skin microcirculation can be investigated using several techniques. Some of them can visualize it directly such as Nailfold Videocapillaroscopy and Sidestream Dark Field Imaging, others show tissue perfusion such as Laser Doppler Flowmetry and Laser Speckle Contrast Imaging.

This mini review covers the following techniques: Nailfold Videocapillaroscopy, Orthogonal polarization Spectral Imaging, Sidestream Dark Field Imaging, Incident Dark field Illumination, Laser Doppler Flowmetry, and Laser Speckle Contrast Imaging. At the end, some clinical applications of skin microcirculation measuring techniques will be mentioned.

## Direct visualization of the microcirculation

### Nailfold videocapillaroscopy (NVC)

The first reports of studies with capillaroscopy date back to 1663 by Johan Christophorous Kolhaus, who was the first clinician to use a primitive microscope to observe the nailfold. More than a 100 years later, Giovanni Rasori (1776_1873) showed, with magnifying glasses, the relationship between conjunctival inflammation and presence of an inextricable knot of capillaries (8, 9).

Nailfold videocapillaroscopy is a simple and reproducible test that can be used in adults and children (10). It is a non-invasive diagnostic method used to study microvascular abnormalities. This technique usually involves an epi-illumination microscope, video camera, computer, and the images can be digitally stored in mp4 format.

The NVC can be used to study the structure of the microcirculatory bed evaluating capillary morphology and diameters and its function by measuring red blood cell velocity (RBCV) at rest. A physiological test can be performed by putting a torniquet on the proximal phalanx of one finger, inflating it above the systolic pressure and making a 1-min ischemia. The release the pressure leads to reactive hyperemia response where maximum red blood cell velocity and time to reach it may be measured.

The NVC exam can be summarized as follows: After 4 h fasting, the subject waits in an acclimatized room with controlled temperature of 24 ± 1°C for 20 min. After this period, the subject sits comfortably in a high chair, and the fourth left finger is placed, at the heart level, on an acrylic platform of a Leica MZFLIII stereoscopic microscope (Wetzlar, Germany) equipped with an epi-illumination system (100 W Xenon lamp). Coupled to this system, there is a video camera Leica DFC365 FX and an HP Z420 workstation (Intel Xeon CPU E5-1620 3.60 GHz, 8.0 GB RAM, 500 GB hard drive) with a 30″ NEC monitor. The finger skin temperature must be kept at 25 ± 2°C and monitored throughout the exam with an YSI Precision 4000 A digital thermometer (Dayton, OH, USA) with the thermistor probe taped within 1 cm proximal to the nailfold. A drop of mineral oil is placed on the nailfold bed to improve image quality by diminishing the divergence of reflected light. A pressure cuff connected to a mercury manometer is placed on the evaluated finger for functional testing of the microcirculation (reactive hyperemia response). FCD (Functional capillary density, the number of capillaries in the microscopic field with flowing red blood cells per unit tissue area) is evaluated using 250× magnification. At a magnification of 680×, we obtain capillary diameters (AFD—afferent, APD—apical, and EFD—efferent; in micrometers), basal RBCV (in mm/s), maximum RBCV after 1 min of arterial occlusion release in the evaluated finger (RBCV_max_; in mm/s) during the reactive hyperemia response, time to reach RBCV_max_ in seconds (TRBCV_max_), and RBCVmax/RBCV. Analyses of three fields of the same periungueal bed are performed: medial, central, and lateral. The average of these measurements is determined to increase exam reliability by reducing measurement variability (11, 12; Figure 1B).

### History of OPS, SDF, and IDF techniques

The Orthogonal Polarization Spectral Imaging (OPS) technique through Cytoscan device (Cytometrics, Philadelphia, PA, USA) was the first hand-held vital microscopy described by Groner et al. (13). This device was revolutionary because until then only surgical preparations or directly nailfold microcirculation could be evaluated through intravital microscopy and nailfold videocapillaroscopy, respectively. With the Cytoscan it was possible to evaluate any microcirculatory bed in any surface including skin and mucosa. After a few years this device has been replaced for a second generation of hand-held microscope with Sidestream Dark-Field Imaging (SDF) technique (14) which is commercially available by two companies: Microscan (Microvision Medical B.V., Amsterdam, the Netherlands) and Capiscope Hand-held Video Capillaroscopy System (HVCS) and Capiscope HVCS (high resolution) (KK technology, Honiton, United Kingdom). The third generation created was Incident Dark-Field Illumintaion (IDF) technique with the Cytocam device (Braedius Medical B.V., The Netherlands) presented in Hutchings et al. (15).

### Orthogonal polarization spectral imaging (OPS)—Cytoscan

**FIGURE 2:**
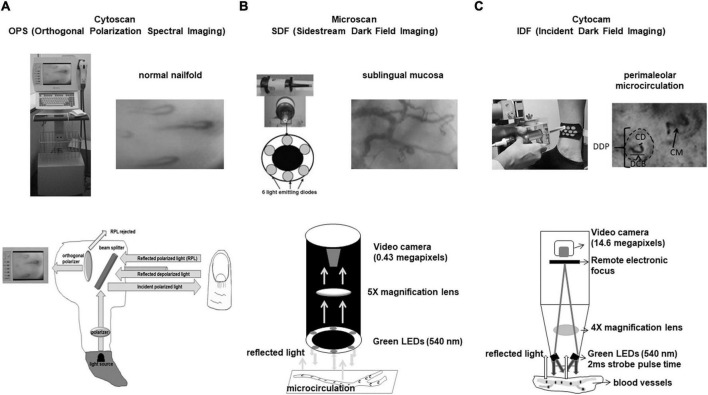
**(A)** Cytoscan (OPS technique). Reflected depolarized light from tissue is used to build flowing red blood cells in grayish background. Here one can see the normal nailfold microcirculation. Capillaries have hair pin format. **(B)** Microscan (SDF technique). Illumination is provided by surrounding central light guide by concentrically placed six light emitting diodes (LEDs) to provide sidestream dark field illumination. Sublingual mucosa microcirculation is shown and we see arterioles, venules, and capillaries very different from nailfold microcirculation where only capillaries can be visualized. **(C)** Cytocam (IDF technique). IDF associated to optical and better visualization of microcirculation enhanced SDF technology. Cytocam uses incident dark field illumination with high-brightness LEDs with very short illumination pulse time of 2 ms. Perimaleolar microcirculation is shown and the following parameters can be evaluated: DDP, diameter of dermal papilla; DCB, diameter of capillary bulk; CD, capillary diameter; CM, capillary morphology.

The OPS technique uses a polarized light with wavelength of 548 nm, where oxy- and deoxyhemoglobin can absorb light equally. A beam splitter projects light to the tissue that penetrates 3 mm into skin. Reflected polarized and depolarized light are analyzed by a polarizer (analyzer) placed orthogonally to the light source. The microcirculatory images are built with depolarized light and a video monitor shows red blood cells in black and light background (13, 16–18; Figure 2A).

### Sidestream dark field imaging (SDF)—Microscan

In the SDF technique, illumination is provided by surrounding a central light guide by concentrically placed 6 light emitting diodes (LEDs) to provide sidestream dark field illumination. The LEDs emit at central wavelength of 530 nm, chosen to correspond to an isosbestic point in the absorption spectra of deoxy- and oxyhemoglobin. The light from microscope illuminates the microcirculation by scattering and reflected light building the microcirculatory environment. Red blood cells (RBC) are imaged as dark moving objects against a white/grayish background. The image quality of RBC is improved by pulsed illumination in synchrony with the CCD frame rate to perform intravital stroboscopy. This maneuver avoids blurring of RBC and vessels (19–21; Figure 2B).

### Incident dark field illumination (IDF)—Cytocam

The IDF technique is a combination of IDF illumination with optical and technical features optimized for visualization of the microcirculation on tissue surfaces. It uses incident dark field illumination with high-brightness LEDs with very short illumination pulse time of 2 ms. The illumination pulses are electronically synchronized for image acquisition producing high penetration and sharp contour visualization of the microcirculation (22–24; Figure 2C).

### Comparison between SDF and IDF

The Microscan (Mic, SDF) has an image size of 720 × 480 and the Cytocam (Cyt, IDF) 2208 × 1648 pixels. The Microscan has lower resolution (micrometers/pixel) and megapixels compared to Cytocam (Resolution: Mic 1.45, Cyt 0.66; Megapixels: Mic 0.43, Cyt 14.6). The frame rate (fps) is 30 with Microscan and 25 with Cytocam while pulse time (ms) and magnification are higher for Microscan compared to Cytocam (Pulse Time: Mic 10, Cyt 2; Magnification: Mic 5, Cyt 4). Given these previously mentioned features, they all contribute to increased contrast of microcirculatory images and increased capillary density detection by Cytocam compared to Microscan (25, 26).

### Parameters obtained by OPS, SDF, and IDF techniques

Microvascular diameters, microvessel morphology, functional capillary density, and red blood cell velocity can usually be measured in organs and mucosa. However, the visualization of skin microcirculation is different, and it has been necessary to create new parameters for its evaluation. In the skin, the dermal papillae with capillaries inside them are visualized (morpho-functional unit of the skin’s microcirculation). Thus, some researchers created new measurement parameters such as dermal papilla, capillary bulk and capillary diameters. It is also possible to calculate the functional capillary density, but it is not possible to measure red blood cell velocity in capillaries that are arranged perpendicular to the skin (27, 28; Figure 2C).

## Tissue perfusion and blood flow analysis

### Laser Doppler flowmetry (LDF)

Laser Doppler flowmetry is a non-invasive technique based on the reflection of light from the laser beam. Light undergoes changes in wavelength (Doppler shift) when it is reflected by red blood cells moving in the microvasculature. A photodiode measures the movement of red blood cells and evaluates red blood cell velocity and concentration expressed by perfusion index. The infrared light from a low-power laser is directed *via* an optical fiber onto the tissue to be studied, and the light scattered back from the tissue is collected by 1 or more other optical fibers and analyzed. The LDF penetrates the skin around 1.5 mm and measures microvascular blood perfusion in arbitrary units (29, 30; Figure 1C). The use of iontophoresis and Laser-Doppler Flowmetry have been widely used to investigate microvascular function in many diseases. Iontophoresis is a technique where charged substances are introduced into the skin by means of a small electrical current. It is based on the principle that a charged molecule migrates under the influence of an applied electric field toward opposite charged electrodes placed on the tissue. Generally, an electrode consists of a conductive sponge, containing the material to be introduced. The other electrode is located at some distance from the first (31). For example, skin vasodilatory response to iontophoretically applied acetylcholine, an endothelium dependent vasodilator and to sodium nitroprusside, an endothelium independent vasodilator.

### Laser speckle contrast imaging

Adequate perfusion is responsible for tissue survival. There are many lasers based optical imaging modalities to assess tissue perfusion and therefore the microcirculation. Laser speckle contrast imaging (LSCI) is an example. This technique is fast, not expensive and relatively simple. It produces 2-D perfusion maps of tissue surfaces and perfusion in arbitrary units. Fercher and Briers reported the first biomedical application of LSCI in. Its method was not on real-time and had limitations such as slow processing time in non-digital systems at that time (32). Currently this technique is performed in real time.

Laser speckle contrast imaging is based on the principle that the backscattered light from a tissue that is illuminated with coherent laser light forms a random interference pattern at the detector, called speckle pattern. With red blood cells movement, fluctuations in this speckle pattern is produced causing blurring of images obtained with an exposure time equal to or longer than the speckle fluctuation time scale (33; Figure 1D). This technique has some limitations as movement of tissue interferes with laser speckle and it is still controversial whether LSCI measures blood flow or red blood cell velocity (34).

### Clinical applications of skin microcirculation imaging techniques

Nailfold videocapillaroscopy has been used to investigate the microcirculation in rheumatological or dermatological diseases such as systemic lupus erythematosus (11, 35), primary Sjögrens’s Syndrome (36), systemic sclerosis (37), and psoriasis (38, 39). As peripheral microcirculation can mirror vascular diseases, important research has been developed with Diabetes Mellitus (40), coronary artery disease (41, 42), and arterial hypertension (43) as well. OPS, SDF, and IDF technique have been used to investigate skin or sublingual microcirculation in chronic venous disease (27) and healthy volunteers (23). Investigation of tissue perfusion performed by LDF and LSCI is also present in the literature. Atherosclerosis, high blood pressure, heart disease, Diabetes Mellitus, and kidney failure have been studied by LDF or LSCI (44). Some diseases have been studied by many methods, for example Systemic Sclerosis by NVC (37), Laser Doppler Flowmetry (45) and LSCI (46) or psoriasis by NVC (38, 39) and LSCI (47).

## Discussion

All techniques presented in this mini review have strengths and limitations. For instance, NVC is limited to nailfold and shows only capillaries. On the other hand, it is non-invasive, easy to use and Capillaroscopy has been included in the new 2013 classification criteria for systemic sclerosis (48). Hand-held vital microscopes (HVM) have been developed with techniques that directly visualize the microcirculation including OPS, SDF, and IDF. These techniques have revolutionized the study of microcirculation in the skin and mucosa limited prior to NVC or biopsies. IDF videomicroscope has demonstrated superior quality of sublingual microcirculatory image acquisition compared to SDF video-microscope (49). The second consensus on the assessment of sublingual microcirculation in critically ill patients with HVM was created by the Cardiovascular Dynamics Section of the European Society of Intensive Care Medicine (ESICM) (50). This conference shows the growing importance of microcirculation studies. Finally, LDF and LSCI complement microscopic studies with emphasis on tissue perfusion. LDF has some limitations: movement artifacts and perfusion in absolute units remains a scientific challenge. Regardless of these limitations, laser Doppler Flowmetry remains a highly sought after technique for microcirculatory blood flow studies (51). Tissue motion is the main problem with LSCI technique. Measured perfusion increased with tissue motion speed. Thus, curved tissue surfaces or with movements should be avoided (52).

## Conclusion and future directions

Although all methods presented here have limitations, all of them can adequately assess the structure and functionality of the microcirculation. Future developments in assessment techniques should involve better resolution of generated images, improvement of objective lenses, reduction of influence of tissue movement for image acquisition and visualization of red and white blood cells without artificial contrasts. These proposed technical improvements may enable the study of inflammatory mechanisms *via* leukocytes and increase knowledge about hemorheology of red blood cells in the cutaneous circulation of humans in a non-invasive way.

## Author contributions

DB created the pictures. Both authors concepted the whole work, drafted the manuscript, contributed to the article, and approved the submitted version.

## References

[1] Holowatz L Thompson-Torgerson C Kenney W. The human cutaneous circulation as a model of generalized microvascular function. *J Appl Physiol.* (2008) 105:370–2. 10.1152/japplphysiol.00858.2007 17932300

[2] Abularrage C Sidawy A Aidinian G Singh N Weiswasser J Arora S. Evaluation of the microcirculation in vascular disease. *J Vasc Surg.* (2005) 42:574–81. 10.1016/j.jvs.2005.05.019 16171612

[3] Holowatz L Kenney W. Local ascorbate administration augments NO- and non-NO-dependent reflex cutaneous vasodilation in hypertensive humans. *Am J Physiol Heart Circ Physiol.* (2007) 293:H1090–6. 10.1152/ajpheart.00295.2007 17483240

[4] Lindstedt I Edvinsson M Evinsson L. Reduced responsiveness of cutaneous microcirculation in essential hypertension–A pilot study. *Blood Press.* (2006) 15:275–80. 10.1080/08037050600996586 17380845

[5] Rossi M Carpi A Galetta F Franzoni F Santoro G. The investigation of skin blood flowmotion: a new approach to study the microcirculatory impairment in vascular diseases? *Biomed Pharmacother.* (2006) 60:437–42. 10.1016/j.biopha.2006.07.012 16935461

[6] Yen A Braverman I. Ultrastructure of the human dermal microcirculation: the horizontal plexus of the papillary dermis. *J Invest Dermatol.* (1976) 66:131–42. 10.1111/1523-1747.ep12481678 1249441

[7] Braverman I. The cutaneous microcirculation. *J Investig Dermatol Symp Proc.* (2000) 5:3–9. 10.1046/j.1087-0024.2000.00010.x 11147672

[8] Cutolo M. *Atlas of Capillaroscopy in Rheumatic Diseases.* Milano: Elsevier (2000).

[9] Cutolo M Smith V. State of the art on nailfold capillaroscopy: a reliable diagnostic tool and putative biomarker in rheumatology? *Rheumatology (Oxford).* (2013) 52:1933–40. 10.1093/rheumatology/ket153 23620555

[10] Ocampo-Garza S Villarreal-Alarcón M Villarreal-Treviño AV Ocampo-Candiani J. Capillaroscopy: a valuable diagnostic tool. *Actas Dermosifiliogr.* (2019) 110:347–52. 10.1016/j.ad.2018.10.018 30851874

[11] Dancour M Vaz J Bottino D Bouskela E. Nailfold videocapillaroscopy in patients with systemic lupus erythematosus. *Rheumatol Int.* (2006) 26:633–7. 10.1007/s00296-005-0033-z 16180000

[12] Costa G Shushanof M Bouskela E Bottino D. Oral L-Arginine (5 g/day) for 14 days improves microcirculatory function in healthy young women and healthy and type 2 diabetes mellitus elderly women. *J Vasc Res.* (2022) 59:24–33. 10.1159/000519428 34784595

[13] Groner W Winkelman J Harris A Ince C Bouma G Messmer K Orthogonal polarization spectral imaging: a new method for study of the microcirculation. *Nat Med.* (1999) 5:1209–13. 10.1038/13529 10502828

[14] Treu C Lupi O Bottino D Bouskela E. Sidestream dark field imaging: the evolution of real-time visualization of cutaneous microcirculation and its potential application in dermatology. *Arch Dermatol Res.* (2011) 303:69–78. 10.1007/s00403-010-1087-7 20972572

[15] Hutchings S Watts S Kirkman E. The cytocam video microscope. A new method for visualising the microcirculation using incident dark field technology. *Clin Hemorheol Microcirc.* (2016) 62:261–71. 10.3233/CH-152013 26484715

[16] Lsvdehabfm K. *Orthogonal Polarization Spectral Imaging.* Basel: Karger (2000).

[17] Harris A Sinitsina I Messmer K. Validation of OPS imaging for microvascular measurements during isovolumic hemodilution and low hematocrits. *Am J Physiol Heart Circ Physiol.* (2002) 282:H1502–9. 10.1152/ajpheart.00475.2001 11893588

[18] Černý V Turek Z Pařízková R. Orthogonal polarization spectral imaging. *Physiol Res.* (2007) 56:141–7. 10.33549/physiolres.930922 16555953

[19] Slaaf D Tangelder G Reneman R Jäger K Bollinger A. A versatile incident illuminator for intravital microscopy. *Int J Microcirc Clin Exp.* (1987) 6:391–7. 3429145

[20] Goedhart P Khalilzada M Bezemer R Merza J Ince C. Sidestream dark field (SDF) imaging: a novel stroboscopic LED ring-based imaging modality for clinical assessment of the microcirculation. *Opt Express.* (2007) 15:15101. 10.1364/OE.15.015101 19550794

[21] Balestra G Bezemer R Boerma E Yong Z Sjauw K Engstrom A Improvement of sidestream dark field imaging with an image acquisition stabilizer. *BMC Med Imaging.* (2010) 10:15. 10.1186/1471-2342-10-15 20626888 PMC2915944

[22] Sherman H Klausner S Cook W. Incident dark-field illumination: a new method for microcirculatory study. *Angiology.* (1971) 22:295–303. 10.1177/000331977102200507 5089888

[23] Aykut G Veenstra G Scorcella C Ince C Boerma C. Cytocam-IDF (incident dark field illumination) imaging for bedside monitoring of the microcirculation. *Intensive Care Med Exp.* (2015) 3:1–10. 10.1186/s40635-015-0040-7 26215807 PMC4512989

[24] Milstein D Romay E Ince C. A novel computer-controlled high resolution video microscopy imaging system enables measuring mucosal subsurface focal depth for rapid acquisition of oral microcirculation video images. *Intensive Care Med.* (2012) 38:S271.

[25] Ocak I Kara A Ince C. Monitoring microcirculation. *Best Pract Res Clin Anaesthesiol.* (2016) 30:407–18. 10.1016/j.bpa.2016.10.008 27931644

[26] van Elteren H Ince C Tibboel D Reiss I de Jonge R. Cutaneous microcirculation in preterm neonates: comparison between sidestream dark field (SDF) and incident dark field (IDF) imaging. *J Clin Monit Comput.* (2015) 29:543–8. 10.1007/s10877-015-9708-5 26021740 PMC4565887

[27] Virgini-Magalhães C Porto C Fernandes F Dorigo D Bottino D Bouskela E. Use of microcirculatory parameters to evaluate chronic venous insufficiency. *J Vasc Surg.* (2006) 43:1037–44. 10.1016/j.jvs.2005.12.065 16678701

[28] Lupi O Semenovitch I Treu C Bouskela E. Orthogonal polarization technique in the assessment of human skin microcirculation. *Int J Dermatol.* (2008) 47:425–31. 10.1111/j.1365-4632.2008.03694.x 18412856

[29] Vongsavan N Matthews B. Some aspects of the use of laser Doppler flow meters for recording tissue blood flow. *Exp Physiol.* (1993) 78:1–14. 10.1113/expphysiol.1993.sp003664 8448007

[30] Zegarra-Parodi R Snider E Park P Degenhardt B. Laser doppler flowmetry in manual medicine research. *J Am Osteopath Assoc.* (2014) 114:908–17. 10.7556/jaoa.2014.178 25429081

[31] De Mul F Blaauw J Smit R Rakhorst G Aarnoudse J. Time development models for perfusion provocations studied with laser-doppler flowmetry, applied to iontophoresis and PORH. *Microcirculation.* (2009) 16:559–71. 10.1080/10739680902956107 19488922

[32] Fercher A Briers J. Flow visualization by means of single-exposure speckle photography. *Opt Commun.* (1981) 37:326–30. 10.1016/0030-4018(81)90428-4

[33] Draijer M Hondebrink E Van Leeuwen T Steenbergen W. Review of laser speckle contrast techniques for visualizing tissue perfusion. *Lasers Med Sci.* (2009) 24:639–51. 10.1007/s10103-008-0626-3 19050826 PMC2701498

[34] Heeman W Steenbergen W van Dam G Boerma E. Clinical applications of laser speckle contrast imaging: a review. *J Biomed Opt.* (2019) 24:1. 10.1117/1.jbo.24.8.080901 PMC6983474 31385481

[35] Monfort J Chasset F Barbaud A Frances C Senet P. Nailfold capillaroscopy findings in cutaneous lupus erythematosus patients with or without digital lesions and comparison with dermatomyositis patients: a prospective study. *Lupus.* (2021) 30:1207–13. 10.1177/09612033211010329 33853419

[36] Aguiar T Furtado E Dorigo D Bottino D Bouskela E. Nailfold videocapillaroscopy in primary sjögren’s syndrome. *Angiology.* (2006) 57:593–9. 10.1177/0003319706293127 17067982

[37] Ruaro B Pizzorni C Paolino S Smith V Ghio M Casabella A Correlations between nailfold microvascular damage and skin involvement in systemic sclerosis patients. *Microvasc Res.* (2019) 125:103874. 10.1016/j.mvr.2019.04.004 30974112

[38] Ribeiro C Siqueira E Holler A Fabrício L Skare T. Periungual capillaroscopy in psoriasis. *An Bras Dermatol.* (2012) 87:550–3. 10.1590/S0365-05962012000400005 22892767

[39] Santhosh P Riyaz N Bagde P Binitha M Sasidharanpillai S. A cross-sectional study of nailfold capillary changes in psoriasis. *Indian Dermatol Online J.* (2021) 12:873–8. 10.4103/idoj.IDOJ_793_20 34934725 PMC8653718

[40] Maldonado G Guerrero R Paredes C Ríos C. Nailfold capillaroscopy in diabetes mellitus. *Microvasc Res.* (2017) 112:41–6. 10.1016/j.mvr.2017.03.001 28274735

[41] Zhito AV Iusupova A Kozhevnikova MV Shchendrygina A Privalova EV. E-selectin as a marker of endothelial dysfunction in patients with coronary artery disease including those with type 2 diabetes mellitus. *Kardiologiya.* (2020) 60:24–30. 10.18087/cardio.2020.4.n1066 32394853

[42] Junqueira C Ferreira E Junqueira A Cyrino F Maranhão P Kraemer-Aguiar L Peripheral microvascular dysfunction is also present in patients with ischemia and no obstructive coronary artery disease (INOCA). *Clin Hemorheol Microcirc.* (2021) 79:381–93. 10.3233/CH-201065 34151847

[43] Penna G Garbero R Neves M Oigman W Bottino D Bouskela E. Treatment of essential hypertension does not normalize capillary rarefaction. *Clinics.* (2008) 63:613–8. 10.1590/S1807-59322008000500008 18925320 PMC2664718

[44] Roustit M Cracowski J. Assessment of endothelial and neurovascular function in human skin microcirculation. *Trends Pharmacol Sci.* (2013) 34:373–84. 10.1016/j.tips.2013.05.007 23791036

[45] Waszczykowska A Goś R Waszczykowska E Dziankowska-Bartkowiak B Jurowski P. Assessment of skin microcirculation by laser Doppler flowmetry in systemic sclerosis patients. *Postepy Dermatol Alergol.* (2014) 31:6–11. 10.5114/pdia.2014.40653 24683391 PMC3952049

[46] Della Rossa A Cazzato M D’Ascanio A Tavoni A Bencivelli W Pepe P Alteration of microcirculation is a hallmark of very early systemic sclerosis patients: a laser speckle contrast analysis. *Clin Exp Rheumatol.* (2013) 31:109–14. 23073231

[47] Schaap M Chizari A Knop T Groenewoud H van Erp P de Jong E Perfusion measured by laser speckle contrast imaging as a predictor for expansion of psoriasis lesions. *Ski Res Technol.* (2022) 28:104–10. 10.1111/srt.13098 34619003 PMC9293292

[48] Ingegnoli F Smith V Sulli A Cutolo M. Capillaroscopy in routine diagnostics: potentials and limitations. *Curr Rheumatol Rev.* (2018) 14:5–11. 10.2174/1573397113666170615084229 28641553

[49] Gilbert-Kawai E Coppel J Bountziouka V Ince C Martin D Ahuja V A comparison of the quality of image acquisition between the incident dark field and sidestream dark field video-microscopes. *BMC Med Imaging.* (2016) 16:10. 10.1186/s12880-015-0078-8 26797680 PMC4722634

[50] Ince C Boerma E Cecconi M De Backer D Shapiro N Duranteau J Second consensus on the assessment of sublingual microcirculation in critically ill patients: results from a task force of the European society of intensive care medicine. *Intensive Care Med.* (2018) 44:281–99. 10.1007/s00134-018-5070-7 29411044

[51] Rajan V Varghese B Van Leeuwen T Steenbergen W. Review of methodological developments in laser doppler flowmetry. *Lasers Med Sci.* (2009) 24:269–83. 10.1007/s10103-007-0524-0 18236103

[52] Zötterman J Mirdell R Horsten S Farnebo S Tesselaar E. Methodological concerns with laser speckle contrast imaging in clinical evaluation of microcirculation. *PLoS One.* (2017) 12:e0174703. 10.1371/journal.pone.0174703 28358906 PMC5373607

